# The effect of catastrophising on health-related quality of life in two chronic somatic illness groups among Hungarian adolescents

**DOI:** 10.1192/j.eurpsy.2024.406

**Published:** 2024-08-27

**Authors:** M. Klein, S. Török, Z. Papp, G. Kökönyei

**Affiliations:** ^1^Doctoral School of Psychology; ^2^ Institute of Psychology, ELTE Eötvös Loránd University; ^3^ Institute of Mental Health; ^4^NAP3.0-SE Neuropsychopharmacology Research Group, National Brain Research Program; ^5^Department of Pharmacodynamics, Faculty of Pharmacy, Semmelweis University, Budapest, Hungary

## Abstract

**Introduction:**

Psychological factors, such as emotional regulation strategies, play a crucial role in the management and care of chronic somatic health conditions among adolescents. In the existing literature, catastrophising has been associated negatively with health-related quality of life in the context of chronic health conditions in general. However, there is limited knowledge about its role in specific illness types.

**Objectives:**

We aimed to evaluate the impact of catastrophising on the health-related quality of life in two distinct illness groups: diabetes and oncology, within a Hungarian sample.

**Methods:**

A cross-sectional study using self-report measures was carried out, involving a total of 273 adolescents (mean age: 14.72 years, SD: 1,82 years; 50.2% females) in the two paediatric samples. The diabetes group consisted of 171 participants, while the oncology group was comprised of 102 individuals. No significant differences were found between the two chronic condition groups in terms of gender (χ2 = 116.51; p = 0.50), or mean age (F(-0.82; 0.77) = 1.66; p = 0.19). The short-version of the Cognitive Emotion Regulation Questionnaire (CERQ-short) was used to assess the cognitive emotional regulation strategies of the children, specifically focusing on the catastrophising subscale. Health-related quality of life was measured using the 4.0 version of the PedsQL, which included subscales for Physical, Emotional, Social, and School Functioning.

**Results:**

A Hayes-moderation analysis with an interaction effect was conducted, controlling for gender, age, and the duration of the illness. A significant interaction effect was observed between catastrophising and the illness groups, impacting School Functioning (F(1, 243) = 4.17; p = 0.04), Physical Functioning (F(1, 245) = 4.67; p = 0.03), Social Functioning (F(1, 245) = 4,23; p = 0.04), and Emotional Functioning (F(1, 245) = 4.20; p = 0.04). The association between catastrophising and health-related quality of life remained stronger in the oncology group.

**Conclusions:**

Catastrophising appears to be a risk factor that affects the quality of life of children facing oncology illnesses. Therefore, addressing catastrophising in interventions tailored to this paediatric illness group may be beneficial.

Keywords: catastrophising, health-related quality of life, adolescents, chronic somatic

This study was supported by the Hungarian National Research, Development and Innovation Office (K143764).

**Disclosure of Interest:**

None Declared

